# MaveDB 2024: a curated community database with over seven million variant effects from multiplexed functional assays

**DOI:** 10.1186/s13059-025-03476-y

**Published:** 2025-01-21

**Authors:** Alan F. Rubin, Jeremy Stone, Aisha Haley Bianchi, Benjamin J. Capodanno, Estelle Y. Da, Mafalda Dias, Daniel Esposito, Jonathan Frazer, Yunfan Fu, Sally B. Grindstaff, Matthew R. Harrington, Iris Li, Abbye E. McEwen, Joseph K. Min, Nick Moore, Olivia G. Moscatelli, Jesslyn Ong, Polina V. Polunina, Joshua E. Rollins, Nathan J. Rollins, Ashley E. Snyder, Amy Tam, Matthew J. Wakefield, Shenyi Sunny Ye, Lea M. Starita, Vanessa L. Bryant, Debora S. Marks, Douglas M. Fowler

**Affiliations:** 1 Bioinformatics Division, Walter and Eliza Hall Institute of Medical Research, Parkville, Australia; 2 Department of Medical Biology, University of Melbourne, Parkville, Australia; 3 Brotman Baty Institute for Precision Medicine, Seattle, USA; 4 Department of Genome Sciences, University of Washington, Seattle, USA; 5 Centre for Genomic Regulation (CRG), The Barcelona Institute of Science and Technology, Barcelona, Spain; 6 University Pompeu Fabra, Barcelona, Spain; 7 Department of Laboratory Medicine and Pathology, University of Washington, Seattle, USA; 8 Immunology Division, Walter and Eliza Hall Institute of Medical Research, Parkville, Australia; 9 Department of Microbiology and Immunology, University of Melbourne, Parkville, Australia; 10 Bioinformatics Group, Department of Computer Science, University of Freiburg, Freiburg, Germany; 11 Department of Computer Science, The Graduate Center, The City University of New York, New York, USA; 12 Seismic Therapeutics, Watertown, USA; 13 Department of Systems Biology, Harvard Medical School, Boston, USA; 14 Department of Obstetrics, Gynaecology and Newborn Health, University of Melbourne, Parkville, Australia; 15 Department of Clinical Immunology & Allergy, The Royal Melbourne Hospital, Parkville, Australia; 16 Broad Institute of Harvard and MIT, Boston, USA; 17 Department of Bioengineering, University of Washington, Seattle, USA

**Keywords:** Multiplexed assays of variant effect, MAVEs, Deep mutational scanning, DMS, Variant classification, Functional genomics

## Abstract

Multiplexed assays of variant effect (MAVEs) are a critical tool for researchers and clinicians to understand genetic variants. Here we describe the 2024 update to MaveDB (https://www.mavedb.org/) with four key improvements to the MAVE community’s database of record: more available data including over 7 million variant effect measurements, an improved data model supporting assays such as saturation genome editing, new built-in exploration and visualization tools, and powerful APIs for data federation and streamlined submission and access. Together these changes support MaveDB’s role as a hub for the analysis and dissemination of MAVEs now and into the future.

## Background

Variation within genomes produces interindividual differences governing a multitude of traits, including many implicated in disease. As DNA sequencing continues to become less expensive and more widely deployed, new human genetic variants are being observed at a staggering pace. Among 800,000 individuals in gnomAD v4 [1], approximately 786 million small variants comprising single nucleotide changes and small deletions/insertions have been identified, of which 16 million are missense variants (i.e., single amino acid changes). In contrast, only 1 million missense variants have been annotated in ClinVar [2] and 88% are currently variants of uncertain significance that cannot be used for clinical decision-making. Understanding how these observed variants, as well as others we will encounter as more individuals are sequenced, impact molecular, cellular, and organismal phenotypes represents a central challenge for genomics [3].

In the past, genetic variants would be tested for functional effects in bespoke assays singly or in relatively low numbers, but more recent technologies have enabled multiplexed assays of variant effect (MAVEs) [4, 5]. In a MAVE, the functional effects of thousands or tens of thousands of variants of a DNA regulatory region, coding gene, untranslated region, or other functional element are simultaneously experimentally determined. To achieve this scale, a large library of variants is made and tested in a pooled fashion, using high-throughput DNA sequencing to read out variant effects (for a detailed description see [6–8]).

The result of a MAVE is a comprehensive variant effect map, which contains the experimentally measured effects of most or all of the possible single nucleotide or missense variants, and may include small insertions and deletions. Variant effect maps have proven exceptionally useful. For example, in genes where germline variants can increase disease risk, variant effect maps can help resolve a large proportion of clinical variants of uncertain significance [9, 10]. Variant effect maps can also be used to probe protein sequence/function relationships [11–21], assist in protein design [22], reveal protein structure [23, 24], elucidate regulatory DNA and gene function by interrogating non-coding sequences [25–28], and train or evaluate variant effect predictors [29–32].

Efforts are now underway to scale up MAVEs to cover a significant fraction of the human genome [33, 34], but realizing their potential requires improved discoverability. In 2019, we created MaveDB [35], a public, open source repository for submitting, sharing, and accessing MAVE data and associated metadata in a standardized, searchable format through an easy-to-use web interface. However, the original version of MaveDB suffered from four key limitations. First, it contained only a small fraction of the data available at the time. Second, data from new multiplexed assay methods such as saturation genome editing [19, 36, 37] were not compatible with the original MaveDB data model. Third, the ability to explore datasets was limited and visualizing data required external tools. Finally, MaveDB was not designed with federation across genomic data resources in mind.

To address those limitations, firstly we have expanded the database content by extensively curating multiplexed assay results and encouraging community contributions, constituting a six-fold increase in the total number of variant effect measurements in the database and an over 30-fold increase in the number of datasets compared to the original publication. As of November 2024, MaveDB contained over 7 million variant effect measurements and 1884 datasets. We have also implemented numerous technical advances and data model improvements. This includes refining and formalizing our variant representation with an emphasis on compliance with established standards like HGVS [38], allowing us to support more diverse types of variants and associated experimental designs, while also improving compatibility with emerging standards like the GA4GH Variant Representation Specification (VRS) [39] that will simplify mapping datasets to reference genomic coordinates. We have updated our data model by adding a new type of record for imputation or the combination of results across multiple assays. We also invested in an improved interface for searching and filtering datasets, as well as adding new automatically generated visualizations. Lastly, we further improved the user experience by adding API-based user uploads aimed at researchers who are submitting large or complex datasets, or engaging in MAVE data production at scale.

## Construction and content

MaveDB is designed to store and distribute multiplexed variant functional data, including scores and associated metadata. Minimally, this consists of a collection of variant effect scores that describe the functional consequences of the nucleotide or amino acid variants, as well as information about the target sequence. The metadata typically includes descriptions of the experimental and data analysis methods and references to information in other databases, such as DNA sequencing reads. Most datasets in MaveDB are from published papers, although this is not required for inclusion.

**Fig. 1:**
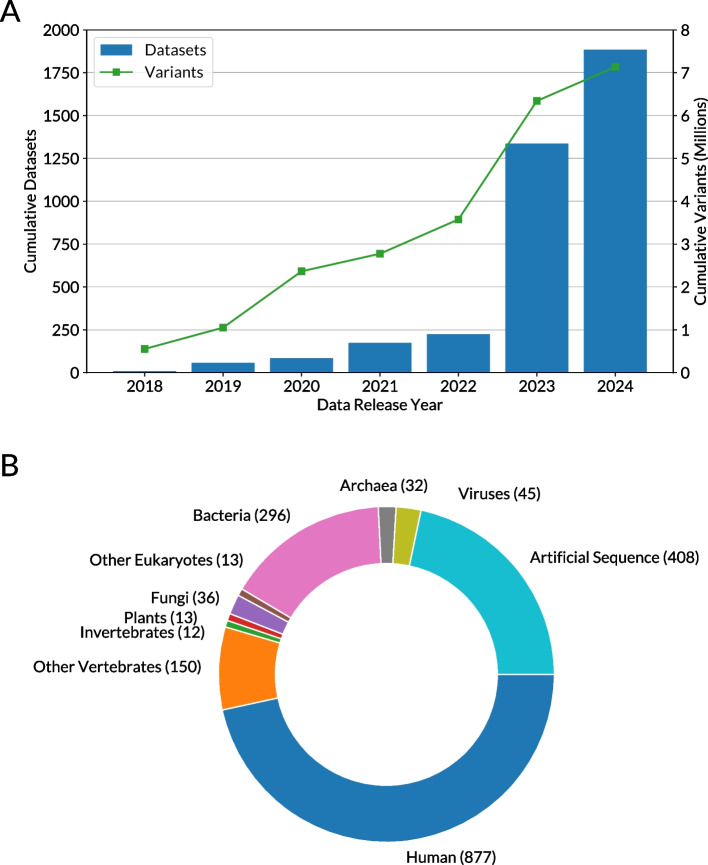
MaveDB contents as of November 2024. **A** Growth of the database by year. The bars show the cumulative number of datasets and the green line shows the cumulative number of variant effect measurements. **B** Diversity of target sequences. NCBI Taxonomy IDs were assigned and grouped according to the categories shown

When the original MaveDB manuscript was published in 2019, only 54 datasets from published MAVEs were included. Thus, we launched a concerted effort to deposit datasets that were not yet included in MaveDB, adding 1228 new datasets containing a total of 3.7 million variant effect measurements. Thanks to this curation and contributions from the community, as of November 2024 MaveDB contained 1884 datasets encompassing 7 million variant effect measurements across diverse targets (Fig. 1).

Our curation team spanned three sites: WEHI and the University of Melbourne in Melbourne, Australia; University of Washington in Seattle, USA; and Harvard University in Boston, USA. We developed a robust process for summarizing heterogeneous experimental results, including training materials, much of which has been incorporated into updated MaveDB documentation available on the website. Key information was extracted from publications and synthesized into a title, short description, abstract, and methods as metadata for each record. Accession numbers for raw sequence data and target sequence identifiers for each dataset were also included. Each curated entry was peer reviewed by at least one other team member to ensure all relevant information was present and accurate before submission to the database. In addition to writing the free text sections and organizing associated metadata, our curation team also formatted scores and related values from published supplemental data.

To make it easier for users to discover MAVE data from publications, in addition to PubMed identifiers, we updated our data model to support bioRxiv and medRxiv preprints and Crossref DOIs. We also store structured metadata for each of these references, including journal or preprint server and all author names, and allow users to search and filter based on this information. MaveDB also now distinguishes between a primary reference, which describes the data contained in the record, and secondary references, which describe methods, key reagents, or software used to generate the data.

MaveDB has a hierarchical structure populated by score set, experiment, and experiment set records. Score set records contain the variant effect scores and associated data columns, such as variance estimates and variant counts, details about the experimental target sequence, and a description of the score calculations. Scores are required, but any number of additional numeric columns can be named by the submitter. Experiment records summarize the assay that was performed and can group multiple score sets, preventing double-counting of assays when raw data is reanalyzed and improving discoverability for users. Experiment set records do not have any data or metadata themselves, but group related experiments, such as multiple assays performed on a single target and described in the same publication. Note that when counting “datasets” above, we counted experiment records since each describes a unique assay on a target.

**Fig. 2:**
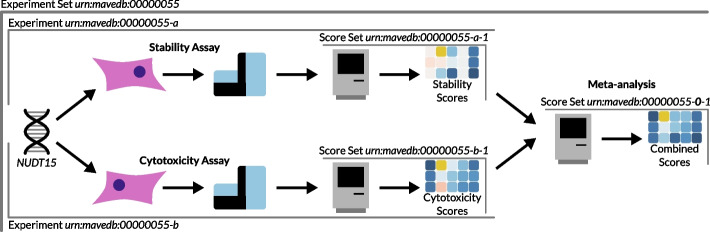
Example of a meta-analysis score set. The cartoon uses a real-world dataset to illustrate the relationship between experiment sets, experiments, score sets, and meta-analysis score sets. The results from two assays performed on the gene *NUDT15* were combined into a resulting “function score” that summarized performance across both assays [40]

To represent scores based on the transformation or combination of existing scores, MaveDB now implements meta-analysis score sets. For example, a dataset that imputes the values of missing scores should be represented as a meta-analysis linked to the pre-imputation score set, ensuring the original scores are preserved and discoverable. Another use case is representing the combination of multiple assay results at the level of the associated scores (Fig. 2).

To improve compatibility with the HGVS Sequence Variant Nomenclature [38], support additional variant types, and enable more robust validation, we implemented MAVE-HGVS, which replaces the previous MaveDB variant representation based on the Enrich2 [41] output format. While packages exist for parsing HGVS [42, 43], they are intended for use in human genetics and rely on sequence database entries that are not always available for multiplexed assay targets. MAVE-HGVS has a reference Python implementation, mavehgvs, used to validate variants uploaded to the database by ensuring variant strings are correctly formatted and consistent with the score set’s target sequence.

To better represent experiments that directly edit the human genome, such as saturation genome editing, we implemented a new way to specify and validate variants. Contributors can now define variants with respect to a transcript accession or a human genome reference, with validation handled by SeqRepo [44] because access to a genome and transcript database is required. This is in contrast to most score set records, which specify their own target sequence and are validated using mavehgvs.

To support current and future developments of the MaveDB platform, particularly API improvements, we have transitioned to a new codebase using FastAPI and Vue.js, replacing the previous codebase that used the Django 1.11 framework. MaveDB now runs as a set of Docker containers orchestrated using Docker Compose, simplifying deployment for the production server as well as for open source developers who wish to contribute to the project. In response to increased usage and demands for greater reliability and future scaling, we have also migrated MaveDB to the cloud using Amazon Web Services.

To promote data federation and the open use of MAVE data globally, we have relicensed nearly all datasets in MaveDB to the Creative Commons CC0 public domain license [45], and now recommend it to submitters. Moving away from the previously recommended but restrictive CC-BY-NC-SA non-commercial license [46] was a result of extensive consultation with maintainers of other biological data repositories as well as the broader MAVE community. This license change combined with the API improvements has allowed us to provide bulk data downloads as described below.

## Utility and discussion

### Web interface

MaveDB features a purpose-built web interface for users to explore and discover datasets as well as upload newly generated or curated datasets. Since the initial launch, the interface has been completely re-implemented using the Vue JavaScript framework. This delivers a more responsive and reactive user experience compared to the previous version of MaveDB, which was based on Django’s HTML templates.

**Fig. 3:**
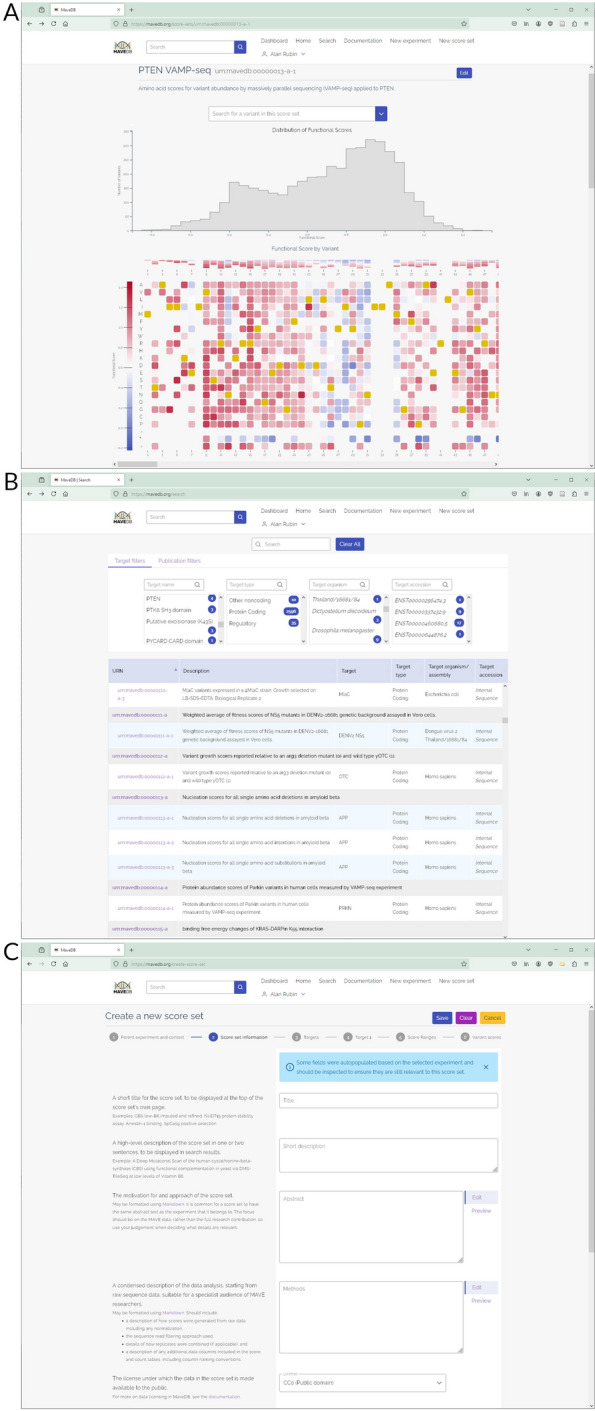
MaveDB web interface screenshots. **A** Score set visualizations. Score set pages now feature automatically generated visualizations, including a score histogram and variant effect heatmap. For non-coding targets, the heatmap is displayed at the nucleotide level. **B** Search page. The interface includes target sequence-based filters at the top, and listings for each matching experiment and its score sets in the main body of the page. MaveDB also supports filtering on publication information such as author via the “Publication filters” tab. **C** Score set creation. Users contributing score sets via the web form can follow this step-by-step workflow with embedded documentation

The score set pages now display automatically generated interactive visualizations for exploration and interpretation, including a score histogram showing the distribution of variant effect scores and a variant effect heatmap (Fig. 3A). The search page has been updated to add categorical filters that encourage exploration of MaveDB data, including publication information such as author or journal (Fig. 3B).

For users who want to contribute data using the web interface, we have overhauled the score set interface to replace the overly-complex single-page form with a guided multi-stage process (Fig. 3C). This simplifies each step of the process and allows for more informative validation and error checking. Guidance for users is now integrated into the form itself, rather than relying entirely on documentation hosted elsewhere on the website.

### Improved API support

The previous version of MaveDB only accepted data via a web form, but the server now also supports data deposition through the REST API using the same logic and validation as the web interface to ensure continuity and data integrity. Using the API to deposit programmatically simplifies submission for some complex experimental designs, such as a series of similar assays that measure variant effects with different small molecules.

To facilitate local validation of datasets, we maintain the MaveDB API code as an installable package on PyPI, the Python Package Index. This allows power users to apply the same validators and data models that are running on the server when preparing datasets for submission. We hope that authors of MAVE analysis pipelines will consider adopting the MaveDB API as an output option.

In addition to serving score set data files identical to those downloadable via the web interface, the API also provides structured data and metadata for individual variants. This feature currently only supports access using MaveDB’s internal variant identifiers, which we are in the process of mapping to more widely used formats [47].

### Bulk data releases

For users who want to access the entirety of MaveDB, we now have an archive of all CC0-licensed data available via Zenodo (see Data availability). It contains a single file in JSON format with all structured metadata for every experiment set, experiment, and score set, as well as a directory of data tables in comma separated value (CSV) format that have the scores and counts for each score set. Archival snapshots increase reproducibility by allowing users to cite a specific version of the database’s contents, and we intend to add complete archives biannually in May and November.

### Recommendations for user uploads

With the introduction of meta-analysis score sets, MaveDB’s hierarchical data model enables more comprehensive provenance tracking for individual variant measurements from a multiplexed assay. We suggest that users upload minimally transformed scores as standard score sets to MaveDB, and create meta-analysis score sets that describe normalization or imputation steps as applicable. This supports other researchers who want to evaluate their own methods or build models that would be sensitive to data normalization.

MaveDB also accepts optional count data for each variant in addition to scores. We strongly encourage submitters to provide this information as it promotes the development of new statistical models for calculating variant scores.

Users should familiarize themselves with the MaveDB hierarchical structure of score set (including meta-analysis), experiment, and experiment set records described above, and try to follow the convention of one experiment per assay and one experiment set per unique target in a study. We recommend that users include the details specified in the MAVE minimum information standards [48] when preparing their textual metadata.

## Conclusions

MAVEs are an important approach for measuring, understanding, and predicting variant effects on a genome-wide scale, but the data must be stored in a stable, standardized fashion along with the metadata required for downstream use. Moreover, MAVE datasets must be readily available and discoverable, and MAVE data must be accessible programmatically. With this 2024 update to MaveDB, we have built on the successes of the initial version of the database and made major strides towards fulfilling these aims.

We made several major improvements to our data model, bolstering our ability to store, standardize, and present heterogeneous MAVE datasets. These changes were made possible by the substantial software engineering effort that went into overhauling the codebase, and we are now better positioned to continue to develop new features like the automatic data visualizations, and respond to innovations in MAVE experimental technologies. Furthermore, we can more easily support specific use cases for MAVE data, including variant effect prediction, drug discovery, and precision medicine.

To increase the amount of information available in MaveDB, we launched a massive curation effort involving hundreds of additional datasets, ultimately populating MaveDB with nearly half of all data published in the literature. In addition, we have seen an encouraging level of engagement from the broader MAVE community, with dozens of international researchers contributing their results of their own accord. We hope that our continued investment in the web interface as well as the API will further encourage prospective users to submit their data, and we thank the many members of the community who have already done so.

## Data Availability

MaveDB source code is available on GitHub [49, 50] and Zenodo [ 51, 52 ]. The version of the MaveDB back-end described here is v2024.4.2 and the version of the MaveDB front-end described here is v2024.4.3. MaveDB is distributed under the AGPLv3 license. mavehgvs source code is available on GitHub [ 53 ] and Zenodo [ 54 ]. The version described here is v0.6.1. mavehgvs is distributed under the 3-Clause BSD license. Notebooks used for generating the panels in Fig. 1 are available on GitHub [ 55 ] and Zenodo [ 56 ]. The version described here is v0.1.0. The notebooks are distributed under the MIT license. The November 2024 MaveDB bulk data download is available from Zenodo [ 57 ]. The dataset depicted in Fig. 2 is available in MaveDB under experiment set urn:mavedb:00000055.
